# Associations of Anxiety, Insomnia, and Physical Activity during the COVID-19 Pandemic

**DOI:** 10.3390/ijerph21040428

**Published:** 2024-04-01

**Authors:** Selene Y. Tobin, Tanya M. Halliday, Kimberley Shoaf, Ryan D. Burns, Kelly G. Baron

**Affiliations:** 1 Department of Health and Kinesiology, University of Utah, Salt Lake City, UT 84112, USA; tanya.halliday@utah.edu (T.M.H.); ryan.d.burns@utah.edu (R.D.B.); 2 Department of Family and Preventative Medicine, University of Utah, Salt Lake City, UT 84112, USA; kimberley.shoaf@utah.edu (K.S.); kelly.baron@utah.edu (K.G.B.)

**Keywords:** anxiety, insomnia, physical activity, bi-directional relationships, COVID-19 pandemic

## Abstract

Purpose: Anxiety, insomnia, and physical activity (PA) are interrelated, but the bi-directional relationships between these three variables are not well understood. Less is known of these relationships in settings of disrupted daily activities and acute stress. This study aimed to characterize and examine relationships between insomnia, anxiety, and PA throughout the first year of the COVID-19 pandemic, when many lifestyle behaviors were disrupted. Methods: Participants comprised a convenience sample of 204 adults (55.4% female; 43.85 ± 15.85 years old) who completed the Generalized Anxiety Disorder Questionnaire (GAD-7), Insomnia Severity Index (ISI), and the International Physical Activity Questionnaire (IPAQ) at three time points through the first year of the COVID-19 pandemic. A cross-lagged panel model was used to evaluate these variables’ concurrent, autoregressive, and cross-lagged relationships across time. Follow-up dynamic panel modeling using maximum likelihood and structural equation modeling was employed. Results: Approximately 64% of participants reported their work/occupation as affected by the pandemic. At baseline, associations between anxiety and insomnia were observed (β-coefficient: 15.87; *p* < 0.001). Insomnia was a positive future predictor of anxiety (ISI time point 2: 7.9 ± 5.6 points; GAD-7 at time point 3: 4.1 ± 4.2 points; β-coefficient: 0.16; *p* < 0.01). No associations were observed between PA and anxiety or insomnia (all *p* > 0.05). Conclusions: Insomnia and anxiety were interrelated, and effects were cross-lagged. These data can inform future work focused on improving anxiety in settings of acute stress and disruptions to daily life, such as changes in occupational structure and stability. Specifically, targeting sleep parameters may be of interest to elicit downstream positive health behaviors.

## 1. Introduction

Since the onset of the COVID-19 pandemic, studies characterizing the profound impacts of the pandemic on daily routines have steadily emerged. While the acute period of severe lockdowns and precautions may be over, the residual effects of the pandemic continue to be revealed. Further, many populations have yet to return to pre-pandemic lifestyles, and there is still much to learn about the downstream impacts of the disruptions to daily life experienced by all. For example, due to the nature of the onset of the pandemic, many people experienced sudden changes to the structure and stability of their careers/occupations, and the recent literature suggests the COVID-19 pandemic’s impact on work trends is likely to extend into the future [1]. Daily activities, such as time spent at work and type of work, are associated with many lifestyle behaviors [2] and overall wellbeing [3]. Some of the many affected lifestyle behaviors include psychological wellbeing, sleep parameters, and physical activity (PA), which have bi-directional relationships between one another as well as with other dimensions of mental and physical health [4,5,6,7,8,9]. Understanding these relationships during a time of global stress provides the opportunity to better understand the potential long-term health consequences of these interrelated behaviors.

Mental wellbeing is a pillar of health that has been reported to be significantly and consistently affected by the pandemic. Anxiety disorder is a mental health condition that includes feelings of worry, nervousness, or unease [10]. The growing literature describing health-related changes during the COVID-19 pandemic shows that anxiety levels have risen sharply upon the onset of the pandemic [11,12]. Prior work demonstrates anxiety to have consequences on indices of sleep and PA levels in some populations [13], which is of particular interest within the context of the shifts in daily behavior experienced during the COVID-19 pandemic.

Relatedly, pandemic-related changes and disruptions to sleep are repeatedly reported throughout the literature [11,14,15,16]. Particularly, reports of fragmented sleep and short sleep duration are of interest as these dimensions of sleep correlate strongly with psychological and physiological health. Dissatisfaction with sleep (sleep maintenance or sleep onset) and symptoms such as daytime sleepiness, impaired attention, and mood disturbances can be characterized as sleep disorder insomnia [17]. Insomnia is strongly related to physical and mental wellbeing; therefore, insomnia symptoms are important to consider within the setting of disruptions to daily activities and acute stress, such as during the first year of the COVID-19 pandemic.

Worldwide, the COVID-19 pandemic also caused profound disruptions in PA behaviors [18]. Furthermore, there is an established relationship between occupation and PA [19]. Changes in one’s occupational status or structure, such as the COVID-19 pandemic, which prompted lockdown measures to curb the rapid transmission of the virus, may influence 24 h movement profiles. However, data before the onset of the COVID-pandemic show that despite the well-documented positive effects of exercise on physical and mental health [20], obtaining a healthy dose of daily movement was already challenging for many. For example, just before the COVID-19 outbreak, the 2019 CDC report shows only 23% of US adults were meeting aerobic and resistance exercise recommendations [21]. Understanding how PA behaviors may have changed during the first year of the COVID-19 pandemic and how these changes related to other parameters of health over time is needed.

Bi-directional relationships between combinations of sleep, anxiety, and PA exist in the literature [22,23]. However, these three variables (sleep, anxiety, and PA) are rarely assessed simultaneously and there are limited data on the effects the disruptions to daily activities elicited by the COVID-19 pandemic had on the relationships between these indices. Much of what has been published on these parameters during the COVID-19 pandemic is cross-sectional, providing only snapshots of these measures in time. Therefore, this study aimed to provide a longitudinal exploratory analysis of the bi-directional associations between anxiety, insomnia, and PA during the first year of the COVID-19 pandemic.

## 2. Methods

A convenience sample of adults aged 18 and older currently residing in Utah was recruited to participate in this remote study. Enrollment was stratified to include a balanced distribution of ages and a representative sample of sex/gender. Additional criteria: Utah residence; able to read and write in English or Spanish; able to access the surveys via smartphone, tablet, or computer; willing to complete the internet-based surveys at three time points. Participants who self-reported severe or unstable illnesses were excluded (e.g., hospitalization in the recent 30 days, current chemotherapy, dialysis, schizophrenia, dementia). We recruited participants using research databases (Research Match), Craig’s List, Reddit, and Facebook, and interested participants completed a preliminary survey to assess eligibility. Research staff called interested participants, reviewed online consent forms, and collected electronic acknowledgments of consent. The Institutional Review Board at The University of Utah approved all study procedures (IRB_00131837).

### 2.1. Study Design

This study was an observational, longitudinal study of mental and physical health behaviors in the first year of the COVID-19 pandemic (March of 2020–February of 2021) on adults in Utah. Specifically, we carried out an analysis of the validated questionnaires that were sent to participants upon enrollment in this study (study time point 1), thirty days after enrollment (study time point 2), and 90 days after enrollment (study time point 3). Questionnaires were sent to participants using REDCap, an electronic data capture tool hosted at The University of Utah [24]. Participants were asked to fill out the questionnaires within 24 h of delivery. Study coordinators monitored compliance daily and followed up with non-compliant participants to ensure the validity of data at a given time point. The overall study also included qualitative interviews and seven days of additional text message-based questionnaires. It is important to note that this manuscript reports on specific outcomes from a selection of the questionnaires collected at each study time point. This reporting strategy allows for a more in-depth exploration of distinct aspects of this study. Additionally, the sample size for this study was calculated based on the daily data collection from the text message questionnaires with the assumption of 20% missing data, resulting in power to observe an effect size of >0.8 for this main outcome.

### 2.2. Outcome Assessments

Upon enrollment, participant demographics were recorded, including age, race, ethnicity, marital status, whether their work was affected by the COVID-19 pandemic (yes/no), household income, and education level. BMI was calculated by self-reported weight in kilograms divided by self-reported height in meters squared (kg/m^2^). Participants completed the following questionnaires:

#### 2.2.1. Insomnia Severity Index

The ISI is an instrument used to assess the severity of nighttime and daytime components of insomnia. Using classical test theory, the psychometric properties of this test were documented, and it has since been examined to identify optimal cut points for case finding in community samples [25]. Consequently, insomnia severity is determined using the following scoring criteria: 0–7 absence of insomnia, 8–14 sub-threshold insomnia, 15–21 moderate insomnia, and 22–28 severe insomnia [25].

#### 2.2.2. Generalized Anxiety Disorder-7

The GAD-7 is a 7-item anxiety scale that has been heavily used throughout the literature. This scale is understood to have good reliability and validity [26]. The following re-established cut points for scores on the GAD-7 were used in this study: 0–4 minimal anxiety, 5–9 mild anxiety, 10–14 moderate anxiety, and 15–21 severe anxiety [26].

#### 2.2.3. International Physical Activity Questionnaire

The IPAQ short form was used to assess participants’ PA behaviors in the recent seven days upon issuing the questionnaire. This validated questionnaire [27] includes questions on the duration and frequency of all PA by intensity (sedentary time, walking, moderate, vigorous). Intensity is quantified as metabolic equivalents (METs), which are an expression of energy cost. One MET is equivalent to an oxygen consumption rate of 3.5 mL/kg/min. This is the average relative oxygen consumption of a person at rest. Typically, light activity is defined as 1.5–3 METs, moderate intensity as 3–6 METs, and vigorous-intensity as >6 METs [28]. For this analysis, the intensity was split into three categories: walking (3.3 METs), moderate exercise (4 METs), and vigorous exercise (8 METs) [29]. The IPAQ prompts participants to provide information on how many days and minutes were spent in each intensity category over the previous seven days. An established equation (shown below) was then used to determine how many MET minutes were spent on moderate-to-vigorous activity per week.

Total MET-min/week = (Walk METs × min × days) + (Mod METs × min × days) + Vig METs × min × days)

### 2.3. Statistical Analysis

Variables were screened for outliers using z-scores and were checked for Gaussian distributions using k-density plots. The primary analysis consisted of a cross-lagged panel model (CLPM) using full information maximum likelihood (FIML). FIML estimation uses all cases within an analysis regardless of any missing data. Thus, missing data were not replaced or imputed; instead, they were handled within the analysis model. CLPM is an analytic approach used to examine both autoregressive and cross-lagged associations for multiple variables observed across time. The potential bi-directional unadjusted associations between the three observed variables (IPAQ, ISI, and GAD-7) were measured at three different time points (time point 1, time point 2, time point 3). A crude CLPM path model was constructed first, followed by a more rigorous model to address the limitations of the CLPM [30]. The first model was a path model, in which all variables were observed (i.e., no latent variables). Specific paths within this CLPM included autoregressive associations, which were the associations between time point 1 to time point 2 and time point 2 to time point 3 for IPAQ, ISI, and GAD-7 to test for the individual variable stability over time. Cross-lagged associations that involved the associations between time point 1 and 2 and between time point 2 and 3 between different variables within the model were also modeled. Cross-lagged associations tested for bi-directionality over time between the IPAQ, ISI, and GAD-7 scores. Standardized covariance coefficients (correlations) at time point 1 (SEMs curved arrows) were also computed. All path coefficients were standardized in the model.

Limitations to the CLPM approach include not effectively controlling for unobserved individual unit effects and difficulty controlling for observed time-invariant covariates. Additionally, there is uncertainty about the treatment of initial conditions using the CLPM approach [31]. Combined, these aspects of the model limit our ability to make causal inferences. To account for these issues, a more rigorous analytical approach was used. Specifically, linear dynamic panel data estimation using maximum likelihood and structural equation modeling (ML-SEM) was employed following the CPLM [32]. The ML-SEM model contained a latent variable, alpha or α, that controlled for individual unit effects similar to random effects CLPM, as suggested by Hamaker et al. [30], thus addressing the instrument variables problem. Under the ML-SEM, the initial conditions were treated as exogenous and therefore did not need to be modeled, and coefficients for the effects of a lagged predictor (x) on a future outcome (y) were constrained to be equal across time. The ML-SEM models were carried out in Stata using the “xtdpdml” command. Directional associations observed from the CPLM were followed up using separate ML-SEM models to determine if the associations were held under this more rigorous analytical approach.

Reporting of the results included the standardized regression coefficients (beta [β]-coefficients) with corresponding 95% confidence intervals. Equation-level goodness-of-fit statistics were also computed and reported as the coefficient of determination (R^2^), or the standardized root mean square residual (SRMR) was calculated and reported, where SRMR scores < 0.08 indicated good model fit [33]. All data are displayed as mean ± standard deviation. The alpha level was set at *p* < 0.05, and analyses were conducted using Stata version 17 statistical software package (StataCorp LLC, College Station, TX, USA).

## 3. Results

Two hundred and four participants (55.4% female; age 43.85 ± 15.85 years old; Table 1) were enrolled in this study and included in this analysis. Approximately 63.7% of the sample reported that their work was affected by the COVID-19 pandemic (Table 1).

Results from the IPAQ showed a skewed distribution. Therefore, the study team determined an upper threshold of 5000 MET-mins, which is at least five times the recommended published 500–1000 MET-mins/week [34]. This decision resulted in 30, 44, and 38 participants’ data from time points 1, 2, and 3, respectively, being replaced as missing data, suggesting that many participants were extreme exercisers or did not accurately report their physical activity. All IPAQ data > 5000 MET-mins were replaced as missing data to utilize the FIML model appropriately. ISI and GAD-7 results were normally distributed without any z-scores over 2.

**Figure 1:**
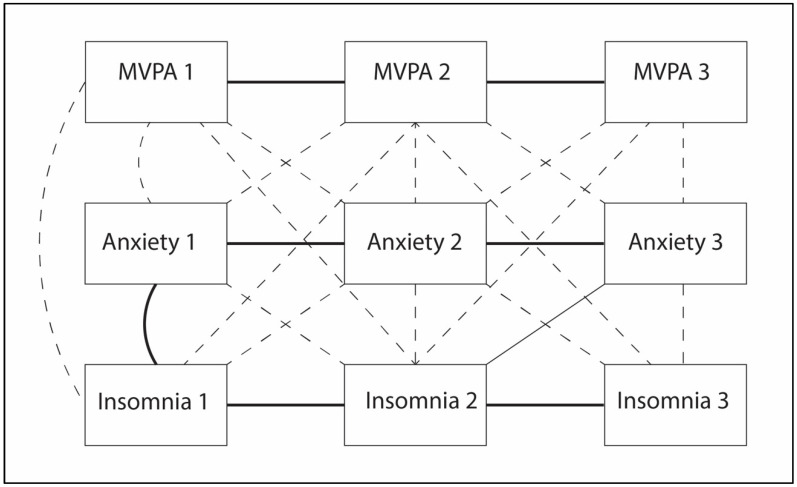
Cross-lagged panel model analysis. Cross-lagged panel model using full information likelihood maximum for associations between insomnia, anxiety, and physical activity across time. Auto-regressive effects showed all variables to be stable across time. Insomnia and anxiety symptoms were correlated at baseline (*p* < 0.001). Significant cross lagged effects between insomnia symptoms at time point 2 and anxiety symptoms at time point 3 were observed (*p* < 0.01). Significant relationships and correlations are denoted by solid lines (thickest solid line: *p* < 0.001; thin solid line: *p* < 0.01); statistically insignificant relationships are denoted by a dashed line. Anxiety symptoms were measured using the Generalized Anxiety Disorder Scale 7; insomnia was measured using the Insomnia Severity Index; MVPA: moderate–vigorous physical activity was measured using the International Physical Activity Questionnaire short form.

The results of the CPLM are reported in Table 2 Auto-regressive effects (within variable associations) within MVPA, ISI, and GAD remained stable over the 3-month enrollment period (time point 1 to time point 2 and time point 2 to time point 3; Figure 1).

There was a significant cross-lagged association (between-variable association) of insomnia symptoms at time point 2 (ISI: 7.9 ± 5.6 points) on anxiety at time point 3 (GAD-7: 4.1 ± 4.2 points; β-coefficient; 0.23; *p* < 0.01; Figure 1, Table 2 and Table 3). Anxiety at time point 2 also displayed an association with MVPA at time point 3 that trended towards significance (GAD-7: 5.2 ± 4.8 points; IPAQ: 1986.3 ± 1245.9 MET-mins/week; β-coefficient; 0.20; *p* = 0.06; Figure 1, Table 2 and Table 3). No other cross-lagged effects were observed (MVPA at time points 1 and 2 with ISI or GAD; ISI and GAD at time point 1 (Figure 1, Table 2 and Table 3).

Standardized covariance coefficients (correlations) were identified between ISI and GAD at baseline (*p* < 0.001; Figure 1). No correlations between MVPA and ISI or GAD were observed (Figure 1, Table 2).

The results of the two ML-SEMs are reported in Table 4. Both models were adjusted for unit effects and time-invariant covariates. Both models were built to follow up on the significant, and nearly significant, associations observed between insomnia and anxiety and anxiety and MVPA, respectively. Insomnia remained a significant positive predictor for future anxiety (*p* = 0.014, Table 4), and displayed significant and positive autoregressive effects across time (*p* < 0.001; Table 4). ML-SEM model fit was acceptable using the anxiety and the insomnia lagged predictor (SRMR = 0.058).

No relationship was observed between anxiety and MVPA (*p* > 0.05; Table 4). There were also no auto-regressive effects of MVPA across time (*p* > 0.05; Table 4). ML-SEM model fit was acceptable for anxiety and the lagged predictor MVPA (SRMR = 0.068).

## 4. Discussion

This study aimed to examine the interrelationships between three key psychological and behavioral variables (insomnia symptoms, anxiety, and MVPA) during the COVID-19 pandemic when disruptions to daily activities were high. Results from our models demonstrated that insomnia and anxiety were interrelated, and effects were cross-lagged, meaning a change in one variable was associated with later changes in the other variable. There was no cross-lagged relationship between insomnia or anxiety with MVPA. Specifically, initial levels of insomnia and anxiety were correlated, and low symptoms of insomnia at time point 2 were related to later reports of low levels of anxiety at time point 3, two months later. Collectively, our results suggest that an association between sleep and anxiety and that insomnia symptoms may relate to future anxiety levels.

Exploration of the relationship between sleep and anxiety is well documented in the literature. There is strong evidence that anxiety contributes to the development of insomnia [8,35]. It is also clear that interventions aimed at improving sleep (i.e., cognitive behavioral interventions, sleep education, yoga, etc.) reduce anxiety [6]. Our study adds to the evidence base of this relationship, particularly that it is bi-directional, and changes in either variable (anxiety or insomnia) can lead to changes in the other in a setting of acute stress.

Our results also provide a longitudinal view of how these variables changed over approximately 3 months during the first year of the COVID-19 pandemic. In our sample, self-reported MVPA was consistently high on average, while insomnia and anxiety remained low and stable for the majority of participants (Table 3). Our cohort reported higher levels of MVPA than the current public health PA guidelines of at least 500 MET-mins/week [28]. These findings contrast with much of the literature assessing MVPA during the early stages of the pandemic [36]. For example, one recent review including empirical studies from Asian, European, and US studies described that decreased PA, mobility, and walking levels linked to the COVID-19 pandemic [37]. Increased sedentary behavior during this time has also been observed in various populations [38]. Instead, our results corroborate with the fewer reports of increased PA via increased outdoor recreation during the first year of the COVID pandemic [37]. This finding is likely because outdoor access in Utah is high, even in suburban and city centers. In 2020, Utah was ranked as the second most active state in the US, with less than 20% of the population considered inactive [21], suggesting that these data align with the typical rates of PA in Utah.

Our findings also revealed low insomnia symptoms that contrast with the large amount of literature reporting increased sleep disturbance during the pandemic [11,14,15,16,39]. Many meta-analyses and systematic reviews have attempted to synthesize the extent of sleep disturbances during the COVID-19 pandemic [40,41]. Cumulatively, the literature shows a 30–75% incidence of sleep disruption across various populations in the first ~2 years of the pandemic [16,42]. Most research examining sleep patterns during the COVID-19 pandemic describes sleep disruptions, poor sleep quality, or difficulty with sleep. However, some reports suggest that some populations experienced improvements in sleep during this time [43,44], which may have been similar to what we observed in our sample. For example, Korman et al. proposed that decreased social pressures lead to relaxed sleep schedules and more sleep opportunity [43]. A previous study describes a well-defined positive relationship between sleep health and PA levels [5]. Perhaps the high levels of PA in our sample help explain the low insomnia symptoms reported in this sample.

Additionally, the low levels of anxiety observed in our sample contrast with much of the published COVID-19-related literature. One systematic review of two hundred and four countries and territories found an average 25.6% increase in anxiety cases [12]. We did not have baseline anxiety levels in our sample and thus cannot draw conclusions regarding potential changes in anxiety levels, which is a common pitfall for COVID-related research. Nonetheless, we did not observe high average levels of anxiety in our data. Previous studies have outlined a clearly defined inverse correlation between PA and anxiety levels [45]. Therefore, it is plausible that the elevated levels of physical activity observed in our sample contribute to the lower self-reported levels of anxiety in this cohort.

Limitations of this study include using a convenience sample, which is likely to be biased toward higher education because of the university setting, and using self-report surveys, which may have been subject to social desirability. In addition, the experience of the pandemic in Utah may have been different than in other areas. For example, the relatively short shutdown period and opportunities for outdoor recreation in Utah are due to a lower population density in comparison to other major cities. We also acknowledge there was a greater amount of missing data on the IPAQ compared to the ISI and GAD-7 due to adding this measure after this study was underway, and due to editing extreme values. Another important consideration of these data is that we reported overall PA and did not have information on the timing and duration of physical activity bouts or the amount of sedentary behavior. Thus, while the relatively high physical activity levels appear favorable for health, we may not have captured long consolidated periods of sedentary behaviors that are known to offset the positive health benefits of PA [46,47,48]. Additionally, participants were enrolled throughout a 1-year period; therefore, our results are not specifically pertinent to one pandemic phase (e.g., early shutdowns vs. later pandemic stressors) and should be conceptualized as capturing a stressful time period overall, with multiple disruptions to work, home, and school routines.

Many early studies assessing people’s health during the COVID-19 pandemic have been cross-sectional in nature [49]. Therefore, an advantage of our study is the use of longitudinal measurements to evaluate change over time and the use of validated measures. Our study adds to the literature about the impact of COVID-19 and the relationships between sleep, anxiety, and PA. Additionally, because the relationships between these behavioral and psychological factors transcend the setting of the pandemic, our results may also be relevant to non-pandemic environments. Thus, our findings have the potential to inform the development of strategies aimed at improving behavioral and psychological health outcomes during acute stress.

## 5. Conclusions

Results from this study demonstrate bi-directional relationships between insomnia and anxiety during a period of acute stress (i.e., changes to work/occupation, etc.) during the first year of the COVID-19 pandemic. Longer-term observational studies and randomized control trials are warranted to better understand these interrelated health behaviors. Overall, our findings can inform future work focused on improving anxiety in settings of acute stress and disruptions to daily life, such as changes in occupational structure and stability. Specifically, we propose targeting sleep parameters, which may be of interest to elicit downstream positive health behaviors.

## Figures and Tables

**Table 1:** Participant characteristics.

Variable	Mean + SD or N (%)	
Age	43.85 ± 15.85 (range, 19–87)	
BMI	27.7 ± 8.1	
Sex/gender	Male	95 (47%)
	Female	103 (50%)
	Transgender Male	3 (1.5%)
	Transgender Female	3 (1.5%)
Education	<12th grade	2 (1%)
	High school graduation or GED	7 (3.5%)
	Some college or associate’s (2-year) degree	42 (21%)
	Bachelor’s (4-year) degree	75 (37%)
	Graduate degree or more	75 (37%)
Has COVID-19 affected your work/occupation?	Yes	130 (64%)
	No	55 (27%)
Household Income	<USD 10,000	10 (5%)
	USD 10,000-USD 25,000	20 (10%)
	USD 26,000-USD 50,000	59 (29%)
	USD 51,000-USD 75,000	23 (11%)
	USD 76,000-USD 100,000	27 (13%)
	>USD 101,000	62 (30%)
Marital Status	Single, never married	58 (27%)
	Married	111 (54%)
	Separated	4 (2%)
	Divorced	21 (10%)
	Widowed	4 (2%)
	Other	3 (1.5%)
Race/Ethnicity	Hispanic or Latino	28 (14%)
	Not Hispanic or Latino	173 (85%)
	American Indian/Alaskan Native	7 (3%)

**Table 2:** Cross lagged panel model estimates for associations between insomnia, anxiety, and PA at all study time points.

Variable 1	Variable 2	β-Coefficient	95% CI	*p*-Value
**Structural**				
MVPA 2	MVPA 1	0.45 ***	(0.23–0.67)	<0.0001
	Anxiety 1	−27.72	(−89.78–34.33)	0.38
	Insomnia 1	14.47	(−35.51–64.45)	0.57
Anxiety 2	MVPA 1	−0.0005	(−0.001–0.0001)	0.11
	Anxiety 1	0.61 ***	(0.48–0.74)	<0.0001
	Insomnia 1	0.05	(−0.06–0.15)	0.37
Insomnia 2	MVPA 1	−0.0003	(−0.0009–0.0004)	0.42
	Anxiety 1	−0.06	(−0.20–0.08)	0.40
	Insomnia 1	0.74 ***	(0.63–0.86)	<0.0001
MVPA 3	MVPA 2	0.59 ***	(0.41–0.76)	<0.0001
	Anxiety 2	51.96	(−2.89–106.81)	0.06
	Insomnia 2	12.25	(−30.00–54.49)	0.57
Anxiety 3	MVPA 2	0.0002	(−0.0002–0.0007)	0.31
	Anxiety 2	0.54 ***	(0.41–0.70)	<0.0001
	Insomnia 2	0.16 **	(0.07–0.267)	0.001
Insomnia 3	MVPA 2	0.0001	(−0.0004–0.0007)	0.63
	Anxiety 2	0.10	(−0.05–0.26)	0.20
	Insomnia 2	0.67 ***	(0.55–0.79)	<0.0001
**Covariates**				
MVPA 1	Anxiety 1	−8.27	(−1273.01–1256.47)	0.98
MVPA 1	Insomnia 1	−951.24	(−2618.46–715.98)	0.26
Anxiety 1	Insomnia 1 *	15.87 ***	(11.26–20.49)	<0.0001

Note: Anxiety symptoms were measured using the Generalized Anxiety Disorder Scale 7; insomnia symptoms were measured using the Insomnia Severity Index; moderate–vigorous physical activity (MVPA) was measured using the International Physical Activity Questionnaire short form; 95% CI stands for 95% confidence interval; bold denotes statistical significance, * *p* < 0.05, ** *p* < 0.01, *** *p* < 0.001.

**Table 3:** Insomnia, MVPA, and anxiety across study time points.

	Questionnaire	N	Avg ± sd	Range
Insomnia Severity Index (ISI)	Time point 1	201	8.5 ± 6.1	0–25
	Time point 2	169	7.9 ± 5.6	0–28
	Time point 3	143	7.0 ± 5.5	0–25
MVPA (MET-min/week)	Time point 1	94	1978.4 ± 1324.8	49.5–4998
	Time point 2	98	2045.9 ± 1382.0	49.5–4982
	Time point 3	91	1986.3 ± 1245.9	33–4776
Generalized Anxiety Disorder Scale (GAD-7)	Time point 1	199	5.5 ± 4.9	0–21
	Time point 2	169	5.2 ± 4.8	0–21
	Time point 3	143	4.1 ± 4.2	0–21

**Table 4:** Standardized estimates from the dynamic panel model using maximum likelihood for the outcome of anxiety and insomnia.

Outcome		β-Coefficient	95% CI	*p*-Value
Anxiety	Lag Anxiety	0.56 ***	0.42–0.69	<0.001
	Lag Insomnia	0.11 *	0.02–0.20	0.014
MVPA	Lag MVPA	0.63	−1.26–2.53	0.51
	Lag Anxiety	30.11	−2445.62–2505.84	0.98

Note: 95% CI stands for 95% confidence interval; bold denotes statistical significance, * *p* < 0.05, *** *p* < 0.001. Anxiety symptoms were measured using the Generalized Anxiety Disorder Scale 7; insomnia symptoms were measured using the Insomnia Severity Index; moderate–vigorous physical activity (MVPA) was measured using the International Physical Activity Questionnaire short form.

## Data Availability

Data supporting reported results can be accessed upon request via emailing Selene Y. Tobin at selene.tobin@utah.edu or Kelly G. Baron and kelly.baron@utah.edu.
